# Liver-specific gene therapy based on self-complementary adeno-associated virus for lysosomal acid lipase deficiency

**DOI:** 10.3389/fphar.2026.1822727

**Published:** 2026-07-01

**Authors:** Ruolan Zhang, Jing Lou, Zheng Jin, Yanmei Ou, Ting He, Hezhi Qu, Yanqun Yang, Xiao Qu

**Affiliations:** 1 School of Life Science and Biopharmaceuticals, Shenyang Pharmaceutical University, Shenyang, Liaoning, China; 2 Shenyang Sunshine Pharmaceuticals, Shenyang, Liaoning, China; 3 Shenzhen Sciprogen Bio-pharmaceuticals, Shenzhen, Guangdong, China

**Keywords:** adeno-associated virus, endoplasmic reticulum stress, gene therapy, lysosomal acid lipase deficiency, mitochondrial dysfunction

## Abstract

**Introduction:**

Lysosomal acid lipase deficiency is a rare, autosomal-recessive disorder caused by inactivating mutations of the lysosomal acid lipase gene and accumulation of cholesteryl esters and triglycerides in lysosomes. Treatment with recombinant lysosomal acid lipase is effective, but involves safety risks and the production of neutralizing antibodies. In the current study, we examined gene therapy with a liver-specific, self-complementary adeno-associated virus 8 (P6-13/rscAAV8) that encodes the human lysosomal acid lipase.

**Methods:**

Two age cohorts of C57BL/6J mice with homozygous lysosomal acid lipase deletion were included. A young cohort (9 weeks of age; n = 8 per dose group, four males and four females) received a single intravenous administration of P6-13/rscAAV8 at 0.6, 2, or 6 × 10^12^ viral genomes per kg (vg/kg). An old cohort (28 weeks of age; n = 4 per dose group, all males) received P6-13/rscAAV8 at 1 or 6 × 10^12^ vg/kg. Control mice received a non-coding vector encoding green fluorescent protein.

**Results:**

In the young cohort, the treatment restored expression of enzyme activity, normalized lipid profiles and body weight, mitigated enlargement of the liver and spleen, and reduced steatosis, inflammation, and fibrosis in the liver. These effects were associated with rescue of autophagic flux and mitochondrial function, as well as reduction of endoplasmic reticulum stress. Notably, the observed effects were much weaker when gene therapy with the same dose was conducted in the old cohort.

**Conclusions:**

These findings suggest that P6-13/rscAAV8, when delivered early enough, may mitigate or even prevent the pathology of lysosomal acid lipase deficiency.

## Introduction

Lysosomal acid lipase (LAL) deficiency is an autosomal-recessive lysosomal storage disease in which inactivating mutations of the gene encoding LAL reduce enzyme activity (to <1% the normal level in Wolman disease, and to 1%–10% the normal level in cholesteryl ester storage disease) (Reiner et al., 2014; Ivashkin and Zharkova, 2017; Pericleous et al., 2017; Carter et al., 2019; Li and Zhang, 2019; Korbelius et al., 2023; Bastos et al., 2024). This deficiency leads to hepatosplenomegaly, liver fibrosis and cirrhosis, elevated levels of low-density lipoprotein cholesterol (LDL-C) and reduced levels of high-density lipoprotein cholesterol (HDL-C) in serum (Reiner et al., 2014; Pericleous et al., 2017; Li and Zhang, 2019; Korbelius et al., 2023; Bastos et al., 2024).

The standard treatment for LAL deficiency is intravenous infusion with recombinant human LAL (Burton et al., 2015; Burton et al., 2022; Rader, 2015; Aigner et al., 2018; Malinová et al., 2020). This treatment is highly effective, but requires infusion once every 2 weeks due to the short half-life (6 min) (Shirley, 2015). Also, the efficacy of the treatment upon repeated treatment is limited by the generation of antibodies, particularly neutralizing antibodies.

Gene therapy (adeno-associated virus encoding the human LAL) has been shown to mitigate hepatosplenomegaly and dyslipidemia in mice lacking both alleles of the endogenous LAL gene (LAL−/−). Target genes carried by self-complementary adeno-associated virus (AAV) enter the cells in a double-stranded form, allowing for immediate translation, but failed to induce strong transgene expression in the liver (Lam et al., 2022). Another system uses a liver-specific promoter from the human gene encoding α1-antitrypsin (Laurent et al., 2025), but enters the cells in a single-stranded form, and thus requires second-strand synthesis before translation (McCarty et al., 2001; McCarty et al., 2003; McCarty, 2008; Buck and Wijnholds, 2020; Li and Samulski, 2020). A third virus system that combines self-complementary design with a liver-specific promoter derived from the α1-antitrypsin promoter failed to show therapeutic efficacy at doses up to 1 × 10^13^ viral genomes per kg (vg/kg) in mice (Lam et al., 2024). Considering the fact that AAV transduces primate and human cells at 50- to 100-fold less efficiently than mouse cells (Nietupski et al., 2011; Colella et al., 2018), more efficient viral constructs are needed.

The therapeutic efficacy of recombinant AAV vectors is governed by capsid-receptor interactions that determine biodistribution and transduction efficiency (Wang et al., 2024). Several AAV serotypes, notably AAV5, AAV8, and AAV9, have shown efficacy for hepatic targeting. In terms of liver biodistribution, AAV5 demonstrates hepatotropic distribution and good safety profile, evidenced by its use in approved therapies like valoctocogene roxaparvovec for hemophilia A and hemophilia B (Zabaleta et al., 2023; Byrne et al., 2025). However, compared to AAV8, AAV5 often requires higher doses to achieve comparable hepatic transduction, as AAV8 exhibits more potent and efficient tropism for hepatocytes (Vercauteren et al., 2016). Regarding transduction efficiency, both AAV8 and AAV9 achieve high-level hepatocyte expression, but AAV9 is characterized by broad tropism, including significant off-target distribution in the heart and central nervous system (Wang et al., 2024). AAV8 is selected in the current study due to its superior liver-targeting efficiency, reduced off-target risk relative to AAV9, and proven efficacy in preclinical models of lysosomal storage disorders (Costa-Verdera et al., 2021; Smith et al., 2023; Khan et al., 2025).

We engineered a hepatotropic AAV8 virus to express the human LAL gene using optimized codons driven by a liver-specific promoter of the thyroxine-binding globulin gene. This system also integrated an alpha mic/bik enhancer to enhance expression. A series of experiments were conducted to examine the potential therapeutic efficacy of this system in mouse model of LAL deficiency. We also investigated the ability of the transgene-encoded enzyme to be secreted by liver cells and internalized into spleen cells after appropriate glycosylation with mannose-6-phosphate, because these steps are essential to the enzyme function (Gomaraschi et al., 2019; Korbelius et al., 2023).

## Materials and methods

### Plasmid cloning and virus production

**FIGURE 1:**
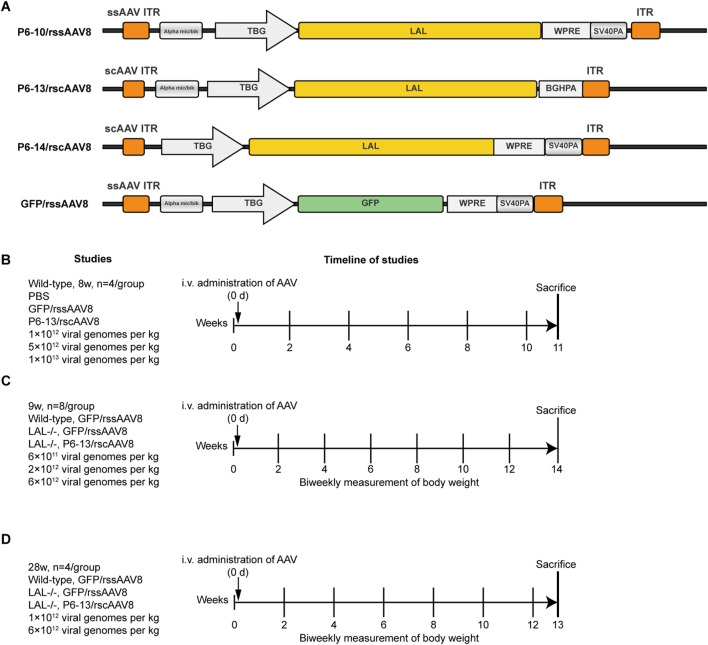
Overall design of the experiments. **(A)** The viral expression constructs. **(B)** Expression and biodistribution of virally transduced lysosomal acid lipase (LAL) in wild-type mice. 8 weeks old mice were treated with a self-complementary virus P6-13/rscAAV8 encoding LAL or a single-stranded virus GFP/rssAAV8 encoding green fluorescent protein (n = 4). **(C,D)** Effects of P6-13/rscAAV8 in mice in **(C)** the young or **(D)** old cohort. The number of mice per condition was 8 (4 each sex) in the young cohort, and 4 (all male) in the old cohort. BGHPA, bovine growth hormone polyadenylation; ITR, inverted terminal repeat; LAL−/−, LAL-deficient; SV40PA, simian virus 40 polyadenylation; TBG, thyroxine-binding globulin; WPRE, woodchuck hepatitis virus post-transcriptional regulatory element.

The full-length cDNA sequence encoding the human LAL and its signal peptide (GenBank accession: NM_001127605.2) was codon-optimized using the codon adaptation index (Plotkin and Kudla, 2011) and synthesized by Genewiz (Suzhou, Jiangsu, China). The transgene also contained the alpha mic/bik enhancer, woodchuck posttranscriptional regulatory element, and simian vacuolating virus 40 polyadenylation signal to terminate transcription. The transgene was subcloned into a recombinant single-stranded AAV8 transfer plasmid containing the thyroxine-binding globulin promoter (P6-10/rssAAV8) (Figure 1A). The green fluorescent protein (GFP) negative control was created by replacing the LAL gene with GFP gene in P6-10/rssAAV8 construct (Figure 1A).

Since the packaging capacity of self-complementary AAV is smaller than that of single-stranded AAV (Li and Samulski, 2020), it was necessary to remove certain elements from the P6-10/rssAAV8 cassette. To explore whether this expression cassette contained an optimal combination of regulatory sequences for translation, P6-14/rscAAV8, a variant similar to P6-10/rssAAV8 but lacked the alpha mic/bik enhancer, was generated (Figure 1A). A second variant, P6-13/rscAAV8, was also similar to P6-10/rssAAV8, but contained bovine growth hormone polyadenylation signal instead of woodchuck posttranscriptional regulatory element and simian vacuolating virus 40 polyadenylation signal (Figure 1A). All plasmid sequences were confirmed through Sanger sequencing (PackGene, Guangzhou, Guangdong, China) prior to packaging. The resulting plasmid was packaged into a virus in 293S cells using a triple-transfection method and purified using affinity and ion-exchange chromatography as described previously (Mietzsch et al., 2020; Khatwani et al., 2021; Florea et al., 2023). Titer of the resulting AAV was determined using quantitative polymerase chain reaction (PCR).

Huh7 cells were infected with one of the three lipase-encoding viruses or the GFP-encoding control virus to assess their ability to drive expression of the human LAL transgene. Huh7 cells (Procell system, Wuhan, Hubei, China) were seeded into 6-well plates at a density of 4 × 10^5^ cells per well in Dulbecco’s modified Eagle medium (Cat: 12430062, Thermo Fisher, Waltham, MA, United States of America) supplemented with 10% fetal bovine serum (Cat: 10091148, Thermo Fisher) and 1% penicillin-streptomycin (Cat: 15140–122, Thermo Fisher). After gentle agitation to ensure even distribution, cells were cultured overnight at 37 °C in a 5% CO_2_ atmosphere to reach approximately 70%–80% confluency prior to infection. For viral transduction, the three lipase-encoding viruses and the GFP-encoding control virus were thawed on ice and diluted in serum-free Dulbecco’s modified Eagle medium to the appropriate working concentrations. Cells were washed once with 1 mL of phosphate-buffered saline (PBS) per well. Subsequently, 1 mL of diluted virus corresponding to multiplicities of infection of 0.1, 0.5, or 2.5 × 10^6^ vg per cell, or serum-free medium (Blank control), was added. Following 24 h of incubation, the viral inoculum was aspirated, and cells were replenished with 1 mL of fresh Dulbecco’s modified Eagle medium containing 5% fetal bovine serum. The medium was replaced with 1 mL of fresh Dulbecco’s modified Eagle medium containing 5% fetal bovine serum at 3 days post-infection. At 7 days post-infection, both culture medium and cell lysates were collected for analysis. For culture medium collection, approximately 1 mL of medium per well was centrifuged at 13000 *g* for 5 min at 4 °C; the clarified supernatant was stored at −70 °C. For cell lysate preparation, cells were washed with ice-cold PBS and lysed on ice for 5 min with 300 μL of ice-cold Pierce® IP Lysis Buffer (Cat: 87787, Thermo Fisher) supplemented with 1 × protease inhibitor cocktail (Cat: 78441, Thermo Fisher). Lysates were centrifuged at 13000 *g* for 10 min at 4 °C, and the medium was stored at −70 °C. Culture medium and cell lysate were assayed for LAL as described below. Following the initial screening (detailed in Results), P6-13/rscAAV8 was chosen for further experiments.

### Secretion of virally delivered LAL and its internalization into uninfected cells

To examine the ability of infected cells to secrete LAL and “pass it onwards” to uninfected cells (Yasuda et al., 2020), Huh7 cells were infected at 0.5 and 2.5 × 10^6^ vg/cell. These higher multiplicities of infection (0.5 and 2.5 × 10^6^ vg/cell) were used to ensure a robust concentration of LAL in the culture medium, which is required to allow for subsequent analysis of secreted LAL glycosylation and its internalization by uninfected K562 cells (Cat: CCL-243, American Type Culture Collection, Manassas, VA, United States of America). Culture medium was harvested at 7 days post-infection and added to cultures of K562 cells that had been seeded into 24-well plates at 2 × 10^5^ cells per well and incubated in minimum essential medium supplemented with 10% fetal bovine serum and 1% penicillin-streptomycin. Some K562 cultures also contained 10 mM mannose-6-phosphate (Cat: CAY-18890–50, Cауmаn, Ann Arbor, MI, United States of America) to outcompete internalization involving the mannose-6-phosphate receptor. The K562 cultures were incubated in conditioned medium for 24 h at 37 °C, washed and lysed, and the intracellular activity of LAL was measured as described below.

### *In vitro* expression of human LAL with and without glycosylation

The codon-optimized transgene encoding the human LAL was subcloned into pcDNA 3.4. A mutation was conducted to convert the three normally glycosylated residues Asn51, Asn80, and Asn300 to Gln (Rajamohan et al., 2020). Huh7 cells were cultured for 20 h, then transfected for 48 h using Lipofectamine™ 3,000 (Cat: L3000150, Thermo Fisher) with a plasmid encoding either the wild-type protein or the triple mutant. After transfection, culture medium and cell lysates were prepared as described in the preceding section for assaying LAL expression and activity.

### Mice lacking LAL

Animal experiments involving wild-type C57BL/6J mice (Figure 1B) were approved by the Institutional Animal Care and Use Committee of Shenzhen Sciprogen Bio-pharmaceutical (Shenzhen, Guangdong, China) (Approval No. P202401). Experiments involving LAL−/− mice (Figures 1C,D) were approved by the Institutional Animal Care and Use Committee of InnoStar Bio-tech (Nantong, Jiangsu, China) (Approval Nos. IACUC-2024-m-943 and IACUC-2025-m-009). Mice with homozygous knockout of the LAL gene (Laurent et al., 2025) were obtained from Shanghai Model Organisms (Shanghai, China). Briefly, mice lacking LAL were generated on a C57BL/6J genetic background using CRISPR/Cas9-mediated genome editing. The target gene was *Lipa* (LAL; ENSMUSG00000024781), and the strategy was designed against transcript *Lipa-202* (ENSMUST00000178114.2). Two guide RNAs (gRNA1: 5′-GCC​TGC​GAG​TAT​AAC​TGA​CTG​GG-3′; gRNA2: 5′-AGT​TAC​CAC​AAG​ACG​GCT​GGA​GG-3′) targeting exon 3 of the *Lipa* gene, along with Cas9 mRNA, were microinjected into fertilized eggs from C57BL/6J mice. This procedure introduced frameshift mutations at the target site via non-homologous end joining repair, leading to a loss-of-function allele. Founder (F0) chimeric mice were identified by PCR and sequencing (Supplementary Figure 1). The line was subsequently expanded and maintained through *in vitro* fertilization and backcrossed for at least six generations to C57BL/6J wild-type mice to achieve a congenic background.​ Homozygous mutant mice from this established line were used for all subsequent experiments in this study. C57BL/6J wild-type mice, also from Shanghai Model Organisms, were used as controls. All mice were housed in a facility free of specific pathogens on a 12-h light-dark cycle with *ad libitum* access to standard food and water.

### Infection and analysis of wild-type mice and mice lacking LAL

To analyze whether and in what tissues the recombinant virus drives expression of the lipase transgene, male wild-type C57BL/6J mice (8 weeks old) received a single intravenous injection of P6-13/rscAAV8 via the tail vein at doses of 0.1, 0.5 or 1 × 10^13^ vg/kg (four mice per condition). Control groups consisted of mice injected with a GFP/rssAAV8 at doses of 0.1, 0.5 or 1 × 10^13^ vg/kg, or with sterile PBS (four male mice per condition). After 11 weeks, the blood, liver, and spleen were harvested. Serum was assayed for LAL activity (see below). Liver tissue was snap-frozen in liquid nitrogen and stored at −80 °C until it was analyzed for the number of viral genomes based on quantitative PCR, expression of LAL mRNA based on reverse transcription-quantitative PCR, as well as activity and glycosylation of LAL (see below). Spleen tissue was frozen, stored, and analyzed in the same way as liver tissue, except that lipase glycosylation was not analyzed.

To analyze the therapeutic effects of viral gene therapy at early stages of LAL deficiency, 9-week old mice (young cohort) lacking the endogenous enzyme received a single intravenous injection of P6-13/rscAAV8 via the tail vein at doses of 0.6, 2 or 6 × 10^12^ vg/kg (four males and four females per condition). Body weight was measured every 2 weeks. After 14 weeks, body composition was measured (see below). Then, blood, liver, spleen, inguinal white adipose tissue (iWAT), interscapular brown adipose tissue (iBAT) and 1-cm segments of the mid-duodenum, jejunum and ileum were collected. Serum was assayed for LAL activity (see below) as well as HDL-C, LDL-C, alanine aminotransferase and aspartate aminotransferase (see below). Liver tissue was snap-frozen in liquid nitrogen, stored at −80 °C. Tissue samples were subjected to comprehensive biochemical and molecular profiling, including: (i) quantification of viral genome copies, and expression and activity analysis of LAL (described below); (ii) assessment of total cholesterol, cholesteryl ester, and triglyceride levels; (iii) evaluation of genes associated with inflammation, fibrosis, and endoplasmic reticulum stress, as well as assessment of autophagy and endoplasmic reticulum stress-related proteins; and (iv) measurement of mitochondrial complex I and IV activities and ATP levels (described below). Spleen tissues were frozen and stored similarly to liver tissues, then analyzed for numbers of viral genomes, activity of LAL, total cholesterol and triglyceride (see below). In parallel, fresh portions of liver and spleen were fixed in 4% formaldehyde (Cat: G1101, Servicebio, Wuhan, Hubei, China) and stained with hematoxylin and eosin, Masson stain to detect collagen or Oil Red O to detect lipids, or immunostained against CD68 as a marker of macrophages (Yang et al., 2022) (see below). Fresh liver (1 mm^3^) was fixed in 2.5% glutaraldehyde (Cat: G1102, Servicebio) for transmission electron microscope (see below).

To analyze the therapeutic effects of viral gene therapy in late stages of LAL deficiency, 28-week old mice (old cohort) lacking the endogenous enzyme received a single intravenous injection of P6-13/rscAAV8 in the tail vein at doses of 1 or 6 × 10^12^ vg/kg (four males per condition). After 13 weeks, serum, liver, spleen, adipose tissue and intestinal segments were analyzed similarly to the procedures for the young cohort.

The abovementioned analyses were conducted in parallel on age-, sex- and litter-matched mice lacking endogenous LAL and on wild-type C57BL/6J mice, all of which were infected with the GFP-encoding virus at a dose of 6 × 10^12^ vg/kg.

### Quantification of viral genomes

Genomic DNA was isolated from liver and spleen tissues using the DNeasy Blood and Tissue kit (Cat: 69504, Qiagen, Hilden, Germany), and DNA concentration was determined using the NanoDrop ONE photometer (Thermo Fisher). Concentrations were converted to viral genomes by linearizing the P6-13/rscAAV8 plasmid with *Sca* I (Cat: ER0432, Thermo Fisher), gel-purifying the linearized DNA using the QIAquick gel extraction kit (Cat: 28704, Qiagen), determining its concentration using the NanoDrop ONE, then diluting it serially into solutions containing 1 µg of genomic DNA from mice lacking endogenous LAL. The serial dilution ranged from 2.5 to 2.5 × 10^7^ vg/μL.

As a complementary measure of the number of viral genomes, quantitative PCR was performed using primers that recognize the inverted terminal repeats of the AAV (forward, 5′-GGA​ACC​CCT​AGT​GAT​GGA​GTT-3′; reverse, 5′-TAA​CGG​CCT​CAG​TGA​GCG​A-3′; Genewiz) and TB Green® Premix Ex Taq™ II (Cat: RR820B, Takara, Kusatsu, Japan). Fluorescence was measured using an ABI 7500 system (Thermo Fisher).

### Reverse transcriptase-quantitative PCR

Total RNA was isolated using the RNeasy Mini Kit (Cat: 74104, Qiagen). RNA purity was examined by determination of the 260 nm/280 nm adsorption ratio (values >2.00). An aliquot (800 ng) was reverse-transcribed using PrimeScript™ RT Master Mix (Cat: RR036A, Takara). The inverse transcription was carried out at 37 °C for 15 min, followed by enzyme deactivation at 85 °C for 5 s. Quantitative PCR was performed as described above using specific primers synthesized by Genewiz (Supplementary Table 1). Expression was quantified using the 2^−ΔΔCt^ method (Livak and Schmittgen, 2001) and normalized to expression of 18S ribosomal RNA.

### Western blotting

Liver tissue was homogenized in RIPA lysis buffer (Cat: 89901, Thermo Fisher) supplemented with 1 × phosphatase inhibitor cocktail. Equal amounts of total protein (20 μg), as estimated by BCA assay (Cat: 23227, Thermo Fisher), were fractionated by 10% or 12% SDS-PAGE gel, transferred to a polyvinylidene difluoride membrane (Cat: 10600023, GE Healthcare Life Science, Amersham Hybond, Germany) using a wet transfer method, incubated with 5% skim milk (Cat: 232100, BD Biosciences, San Jose, CA, United States of America) in 1 × tris buffered saline with Tween 20 (TBST; Cat: 60145ES76, YEASEN, Shanghai, China) for 1 h at room temperature, and then incubated with a primary antibody against LAL (dilution 1:2000; Cat: ab154356, Abcam, Cambridge, United Kingdom), SQSTM1/p62 (p62; 1:1000; Cat: 5114, Cell Signaling Technology, Danvers, MA, United States of America), microtubule-associated protein one light chain three beta (LC3B; 1:1000; Cat: 83506, Cell Signaling Technology), C/EBP homologous protein (CHOP; 1:1000; Cat: 2895, Cell Signaling Technology), eukaryotic translation initiation factor 2 subunit alpha (eIF2α; 1:1000; Cat: 9722, Cell Signaling Technology) or phospho-eIF2α (1:1000; Cat: 9721, Cell Signaling Technology) at 4 °C overnight. Following primary antibody incubation, the membranes were washed in 1× TBST working buffer solution for three times, incubated with a horseradish peroxidase-tagged goat anti-rabbit IgG (H + L) or an anti-mouse IgG (H + L) (Cat: 33201ES60; Cat: 33101ES60, YEASEN) for 1 h at room temperature. After three washes with TBST (5 min each), the membranes were visualized using Pierce™ ECL Western blotting Substrate (Cat: 32209, Thermo Fisher) and analyzed using an iBright Imaging System (Thermo Fisher).

For reprobing, the membranes were washed once with 1× TBS working buffer solution (Cat: 60157ES10, YEASEN) for 5 min at room temperature with gentle shaking, treated with 10 mL of Restore™ PLUS Western blot Stripping Buffer (Cat: 46430, Thermo Fisher) for 10 min at room temperature, followed by three washes with 1× TBS working buffer solution for 5 min each. After stripping, the membranes were re-blocked with TBST containing 5% skim milk at room temperature for 45 min, and washed three times with TBST (5 min each). To assess loading equivalence, the membranes were incubated with a primary antibody against α-tubulin (1:2000; Cat: HRP-66031, Proteintech, Wuhan, Hubei, China) for *in vitro* samples, or heat-shock protein 90 (HSP90; 1:2000; Cat: 13171-1-AP, Proteintech) for *in vivo* tissue lysates, at room temperature for 2 h. The membranes were then washed three times with 1 × TBST, incubated with a HRP-conjugated secondary antibody for 1 h at room temperature, washed again with TBST (3 × 5 min), and visualized using an ECL substrate and imaging system.

### Analysis of the glycosylation state of virally encoded LAL

Liver lysate from infected mice or culture medium from infected Huh7 cells, each containing 40 µg of total protein, were either mock-treated or preincubated for 1 h at 37 °C with Endo H (Cat: P0702L, New England Biolabs, Ipswich, MA, United States of America) or PNGase F (Cat: P0704L, New England Biolabs). Samples were then resolved by 12% SDS-PAGE, transferred to polyvinylidene difluoride membranes and analyzed by Western blotting as described above.

### *In vitro* assay of LAL activity

LAL activity was measured as previously described (Hamilton et al., 2012) using 4-methylumbelliferyl palmitate (Cat: M-690–500, Gold Biotechnology, St. Louis, MO, United States of America) as substrate. Briefly, protein (0.1–1 µg) or serum (0.1–1 µL) were diluted with solution containing 0.345 mM 4-methylumbelliferyl palmitate, 90.9 mM sodium acetate (pH 4.0), 1% (v/v) Triton X-100 and 0.0325% (w/v) cardiolipin (Cat: C0563, Sigma-Aldrich, Darmstadt, Germany) to a final volume of 90 µL. Finally, 10 µL of the LAL inhibitor lalistat 2 (30 μM; Cat: SML2053, Sigma-Aldrich) or the solution (pH 4.0) was added. The reaction was allowed to proceed at 37 °C for 21.5 h in the dark, and was terminated by adding 200 μL of 150 mM EDTA (pH 11.5). Fluorescence was measured on a Synergy LX plate reader (BioTek Instruments, Minneapolis, MN, United States of America) at an excitation wavelength of 360 nm and emission wavelength of 460 nm. The difference in fluorescence between reactions containing lalistat 2 or not was converted to enzyme activity based on a standard curve prepared using 4-methylumbelliferone (Cat: M-499–1, Gold Biotechnology) at concentrations from 0 to 33.3 µM. All assays were performed in triplicate.

### Histology and immunostaining

Tissues were fixed in 4% formaldehyde, embedded in paraffin wax, sectioned to a thickness of 4 μm, and stained with hematoxylin-eosin (Cat: G1076, Servicebio). In parallel, sections were stained with Masson stain using a commercial kit (Cat: G1006, Servicebio) or processed for immunohistochemistry or immunofluorescence. For immunohistochemistry, sections were deparaffinized, rehydrated, and incubated overnight at 4 °C with an anti-CD68 primary antibody (1:500; Cat: GB11067, Servicebio). After washing three times with PBS on a shaker (5 min per wash), sections were incubated for 1 h at room temperature with a horseradish peroxidase-conjugated anti-rabbit IgG secondary antibody (1:200; Cat: GB23303, Servicebio), washed again as above, and developed with a DAB substrate kit (Cat: G1212, Servicebio). The reaction was stopped by rinsing with distilled water. Sequential tyramide signal amplification (TSA) immunofluorescence was used to examine the localization of LAL and CD68. After antigen retrieval in Tris-EDTA buffer (pH 8.0) and bovine serum albumin blocking, tissue sections were incubated overnight at 4 °C with a rabbit anti-LAL primary antibody (1:500; Cat: NBP1-54155, Novus, Centennial, CO, United States of America), followed by incubation with an HRP-conjugated S-Vision polymer secondary antibody (Cat: G1302, Servicebio) and signal development with iF555-Tyramide (red fluorescence; Ex/Em: 557/570 nm; Cat: G1233, Servicebio). The antibody complex was then eluted using an antibody elution solution (Cat: G1266, Servicebio) to prevent cross-reactivity. Subsequently, the same sections were incubated overnight at 4 °C with a rabbit anti-CD68 primary antibody (1:1000; Cat: GB113109, Servicebio), followed by the same HRP-conjugated secondary antibody and signal development with iF488-Tyramide (green fluorescence; Ex/Em: 491/516 nm; Cat: G1231, Servicebio). Finally, nuclei were counterstained with DAPI (Cat: G1012, Servicebio), and autofluorescence was quenched. Sections were mounted using an anti-fade mounting medium. Immunofluorescence images were captured using a Nikon Eclipse C1 fluorescence microscope (Nikon Instruments, Tokyo, Japan). For Oil Red staining, tissue sections were fixed in 4% formaldehyde. After being stained with Oil Red O (Cat: G1015, Servicebio) for 10 min and washed with 60% isopropanol, the sections were couterstained with hematoxylin. Stained or immunostained sections were visualized under a light microscope (SWE-CX63, Servicebio).

Two independent observers blinded to experimental groups quantified inflammation and ballooning degeneration in liver sections after staining with hematoxylin-eosin, inflammation and white pulp injury in spleen sections after staining with hematoxylin-eosin, or collagen deposition in Masson-stained liver sections using a scale from 0 (absent/normal) to 4 (severe/diffuse). Two independent observers quantified immunostaining against CD68 by multiplying staining intensity score (0, none; 1, light yellow; 2, brownish yellow; 3, dark brown) by the percentage of positive cells (0, <5%; 1, 5%–25%; 2, 26%–50%; 3, 51%–75%; 4, >75%). The surface area positive for Oil Red O staining was quantified using Aipathwell® v2 software (Servicebio).

### Transmission electron microscopy

Fresh liver (1 mm^3^) was fixed in 2.5% glutaraldehyde, sliced into ultrathin sections, and examined with an HT7700 electron microscope (Hitachi, Tokyo, Japan) (Dutta et al., 2021). Five non-overlapping micrographs were acquired per sample at magnifications from 2500 × to 10000 × . Mitochondrial area (μm^2^ per mitochondrion) was measured manually using ImageJ software (National Institutes of Health, Bethesda, MD, United States of America).

### Body composition measurement

Body composition (including total body fat, lean mass and fluid) was examined in wake animals using a small animal magnetic resonance imaging (MRI) scanner (Minispec LF50 body composition analyzer, Bruker, Billerica, MA, United States of America) (Shu et al., 2023). Mice placed in a plastic holder without sedation/anesthesia for MRI analysis. Each scan took about 2 min.

### Other analyses

Total lipids were extracted from liver and spleen tissues using a commercial kit (Cat: ab211044, Abcam). Triglyceride level was quantified using a triglyceride assay kit (Cat: A110-1-1, Nanjing Jiancheng Bioengineering Institute, Nanjing, Jiangsu, China). Absorbance was measured at 500 nm on the Synergy LX plate reader. Total cholesterol and cholesteryl ester levels were quantified using Amplex red cholesterol and cholesteryl ester assay kits (Cat: S0211, Beyotime Biotechnology, Shanghai, China), with absorbance measured at 570 nm. Levels of HDL-C, LDL-C, alanine aminotransferase and aspartate aminotransferase in serum were measured on a clinical analyzer 7180 (Hitachi).

Mitochondrial complex I activity in liver tissues was determined using a commercial kit (Cat: BC0515, Solarbio, Beijing, China), with absorbance measured at 340 nm. Complex IV activity was measured using a separate commercial kit (Cat: BC0945, Solarbio), with absorbance measured at 550 nm. Liver ATP level was quantified using a commercial luminescence assay kit (Cat: G4309, Servicebio).

### Statistical analysis

Statistical analyses were performed using SPSS version 27 (IBM Corp., Armonk, NY, United States of America). Normality was assessed using the Shapiro-Wilk test, and homogeneity of variances was evaluated using Levene’s test. For two-group comparisons, independent samples *t*-tests were applied. For multi-group comparisons, data meeting assumptions of normality and homogeneity of variance were analyzed using one-way ANOVA followed by Dunnett’s *post hoc* test. When data were normally distributed but variances were unequal, Welch’s *t*-test was used. For non-normally distributed data, the Kruskal–Wallis test was employed, followed by exact Mann-Whitney *U* tests. Body weight at the final experimental time point was treated as a cross-sectional endpoint and analyzed under the same framework. To account for multiple testing, a Bonferroni correction was applied across the number of planned comparisons. Reported p values are Bonferroni-adjusted (p_adj = p × number of comparisons). Two-tailed p_adj <0.05 was considered statistically significant.

## Results

Among the three AAV vectors that express LAL (see Methods), P6-13/rscAAV8 had the highest expression level, based on analyses of secreted and intracellular enzyme (Supplementary Figure 2). P6-13/rscAAV8 was then tested in healthy wild-type mice to examine LAL expression in the liver (Figure 1B), and in a young (Figure 1C) and old cohort of LAL−/− mice (Figure 1D) to examine the potential efficacy. A GFP-encoding single-stranded AAV was used as a negative control.

### Expression and biodistribution of LAL driven by P6-13/rscAAV8 in healthy mice

**FIGURE 2:**
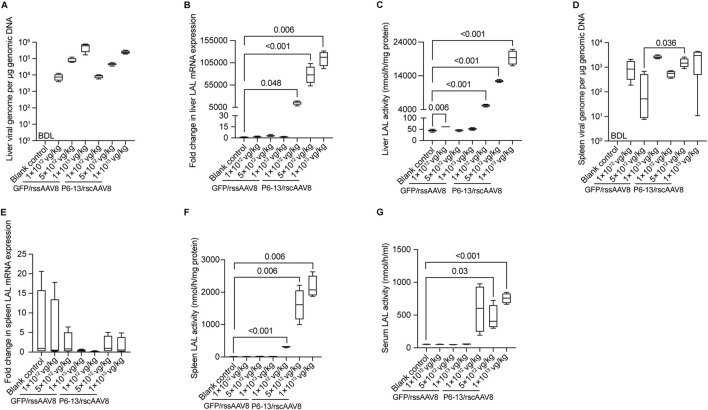
Expression and biodistribution of virally transduced lysosomal acid lipase (LAL) in wild-type mice. Mice were treated as shown in Figure 1B. **(A–G)** Wild-type mice received a single intravenous injection of P6-13/rscAAV8 or GFP/rssAAV8 at 0.1, 0.5 or 1 × 10^13^ viral genomes per kg (vg/kg) (depicted on plots from left to right) and euthanized 11 weeks later. A group of wild-type mice receiving PBS injection was included as a blank control. **(A-C)** Analyses of liver tissue (n = 4). **(D–F)** Analyses of spleen tissue (n = 4). **(G)** Analysis of serum (n = 4). Statistical comparisons were performed against the PBS control group, except for panels displaying viral genomes **(A,D)**, where GFP/rssAAV8 was compared to P6-13/rscAAV8 since the values in the control group were below the detection limit (BDL). All p values are Bonferroni-adjusted for multiple comparisons.

Administration of P6-13/rscAAV8 into wild-type mice increased, in a dose-dependent manner, the number of viral genomes, the level of mRNA encoding LAL, and its activity in the liver after 11 weeks. The number of viral genomes was similar for P6-13/rscAAV8 and the GFP-encoding control single-stranded virus (Figure 2A). P6-13/rscAAV8 increased lipase mRNA levels to >10^4^-fold relative to the PBS-treated blank control (p < 0.048; Figure 2B). No significant differences were observed between the PBS group and the control virus group. P6-13/rscAAV8 increased LAL activity to >10^2^-fold relative to the PBS control group (p < 0.001; Figure 2C).

In the spleen, however, infection with either type of the virus led to similar numbers of viral genomes (Figure 2D), and both virus groups showed similar LAL mRNA levels relative to the PBS group (Figure 2E). Nevertheless, the activity of LAL was >20-fold higher in the P6-13/rscAAV8 group than in the PBS control group (p = 0.006; Figure 2F), with no significant differences between the PBS and control virus groups. Similarly, in serum, P6-13/rscAAV8 treatment led to LAL activity levels approximately 10-fold higher than the PBS control group (Figure 2G), with no significant differences between the PBS and control virus groups. These results suggest that P6-13/rscAAV8 drives production of LAL in the liver, which is then secreted into the circulation and internalized by spleen cells.

### Effects of P6-13/rscAAV8 in the liver of LAL knockout mice

P6-13/rscAAV8 increased the number of viral genomes, level of lipase-encoding mRNA and lipase activity in liver in a dose-dependent manner in both the young and old cohorts (Supplementary Figure 3A-C). Magnitude of LAL increase was larger in the young cohort (∼13- to 81-fold relative to healthy controls) than in the old cohort (∼25- to 63-fold). Western blotting of liver lysates confirmed the expression of the virally delivered transgene, showing a band at the molecular weight expected for mature human LAL (Supplementary Figure 3D).

**FIGURE 3:**
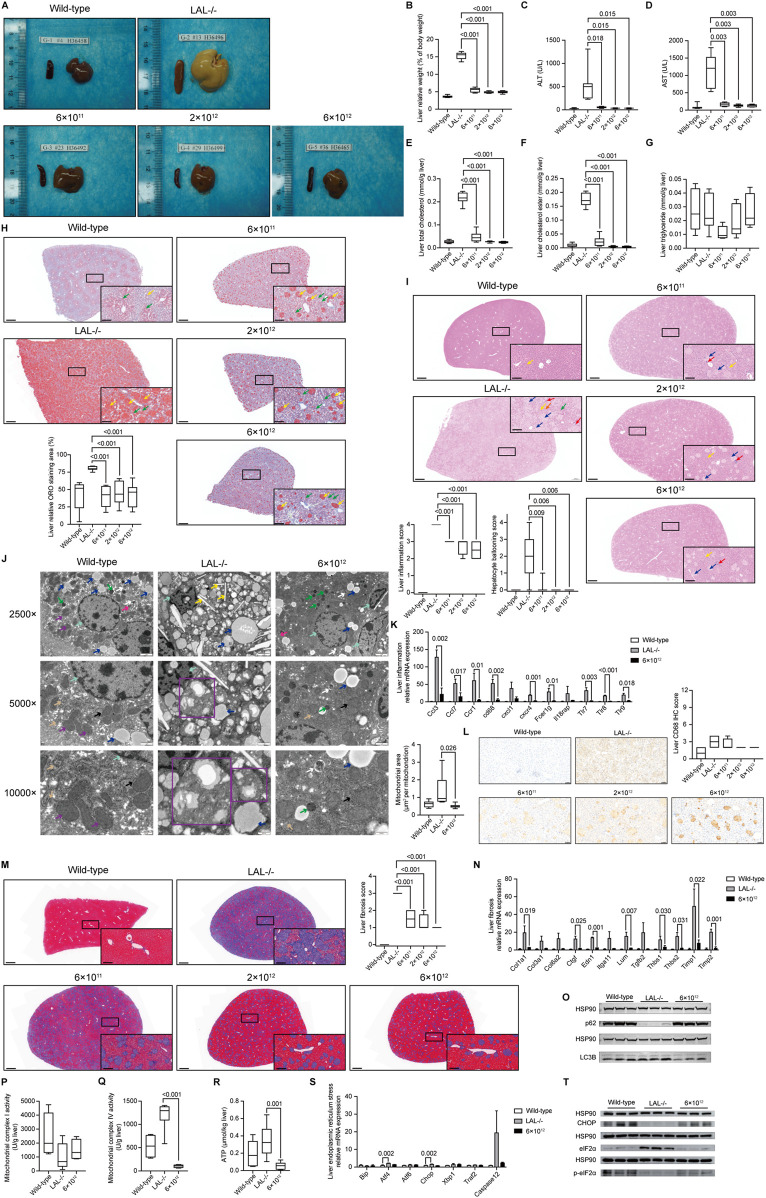
Effects of P6-13/rscAAV8 in livers of young cohort. Mice were treated as indicated in Figure 1C. **(A)** Gross morphology of the liver and spleen (n = 8). **(B)** Relative liver weight, expressed as a percentage of body weight (n = 8). **(C,D)** Levels of alanine aminotransferase (ALT) and aspartate aminotransferase (AST) in serum (n = 8). **(E–G)** Levels of total cholesterol, cholesterol ester and triglyceride in the liver (n = 8). **(H,I,M)** Liver sections were stained with **(H)** Oil Red O (ORO), **(I)** hematoxylin-eosin or **(M)** Masson stain (n = 8). In ORO-strained sections, green arrows denote hepatocyte steatosis and yellow arrows denote macrophage lipid accumulation. In the hematoxylin-eosin-stained sections, yellow arrows denote hepatocyte steatosis, orange arrows denote hepatocyte ballooning, blue arrows denote macrophage hyperplasia with intracytoplasmic lipid vacuoles, red arrows denote inflammatory cell infiltration, and green arrows denote fibrosis. The black boxes (*insets*) are higher-magnification images. Scale bars, 500 μm or 50 μm (inset). **(J)** Representative transmission electron micrographs of liver tissue (n = 5). Magnification is shown at the *left*. Blue arrows denote lipid droplets; yellow arrows, cholesterol crystals; red arrows, glycogen granules; green arrows, lysosomes; pink arrows, autophagosomes; white arrows, autolysosomes; purple arrows, mitochondria (the purple box outlines swollen, lipid-accumulated mitochondria); brown arrows, endoplasmic reticulum; black arrows, mitochondria-associated endoplasmic reticulum membranes; and cyan arrows, the nucleus. Scale bars, 2 μm (2,500 ×), 1 μm (5,000 ×) or 500 nm (10000 ×). **(K,L,N,S)** Expression of relevant markers in liver tissue, including **(K)** genes associated with inflammation, **(L)** the macrophage marker CD68, **(N)** genes associated with fibrosis, or **(S)** genes associated with endoplasmic reticulum stress (n = 8). Expression was assessed in terms of levels of the corresponding mRNAs, except CD68, which was assessed through immunostaining. Scale bar, 50 μm. **(O)** Representative Western blotting against the autophagy markers sequestosome-1 (p62) and microtubule-associated protein one light chain 3b (LC3B) in liver lysates (n = 3). **(P-R)** Quantification of mitochondrial complex I activity, complex IV activity, and ATP levels in liver tissues (n > 3). **(T)** Representative Western blotting against the endoplasmic reticulum stress proteins C/EBP homologous protein (CHOP), eukaryotic translation initiation factor 2 subunit alpha (eIF2α) and phospho-eIF2α (p-eIF2α) in liver lysates (n = 3). Statistical comparisons were performed against the LAL−/− control group. All p values are Bonferroni-adjusted for multiple comparisons. IHC, immunohistochemistry; LAL−/−, lysosomal acid lipase activity-deficient.

**FIGURE 4:**
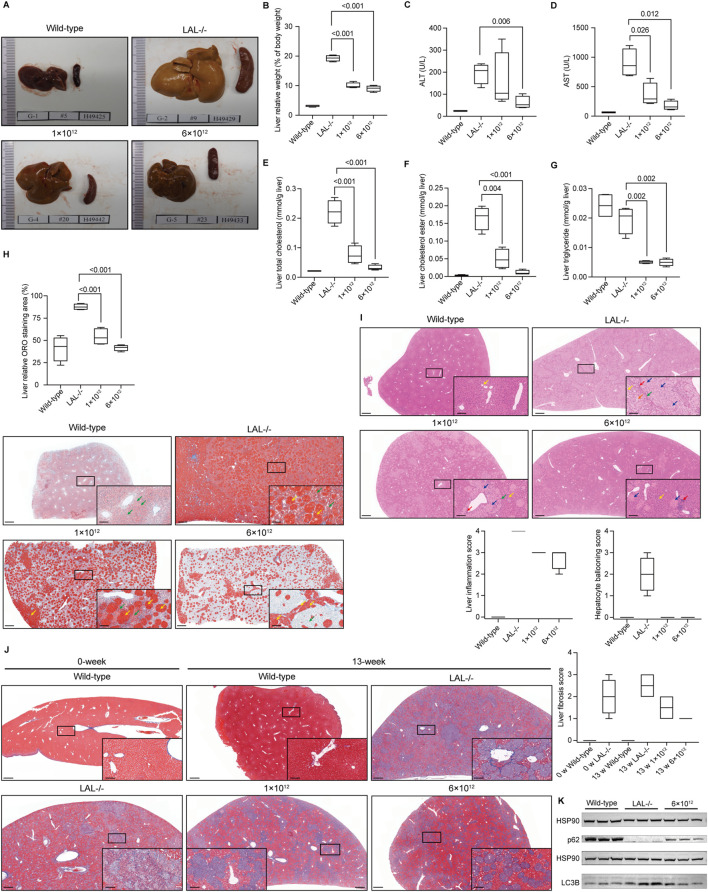
Effects of P6-13/rscAAV8 in livers of old cohort. Mice were treated as indicated in Figure 1D. **(A)** Gross morphology of the liver and spleen (n = 4). **(B)** Relative liver weight, expressed as a percentage of body weight (n = 4). **(C,D)** Levels of alanine aminotransferase (ALT) and aspartate aminotransferase (AST) in serum (n = 4). **(E–G)** Levels of total cholesterol, cholesterol ester and triglyceride in the liver (n = 4). **(H–J)** Liver sections were stained with **(H)** Oil Red O (ORO), **(I)** hematoxylin-eosin or **(J)** Masson stain (n = 4). In ORO-strained sections, green arrows denote hepatocyte steatosis and yellow arrows denote macrophage lipid accumulation. In the hematoxylin-eosin-stained sections, yellow arrows denote hepatocyte steatosis, orange arrows denote hepatocyte ballooning, blue arrows denote macrophage hyperplasia with intracytoplasmic lipid vacuoles, red arrows denote inflammatory cell infiltration, and green arrows denote fibrosis. The black boxes (*inset*) are higher-magnification images. Scale bars, 500 μm or 50 μm (inset). **(K)** Representative Western blotting against the autophagy markers sequestosome-1 (p62) and microtubule-associated protein 1 light chain 3b (LC3B) in liver lysates (n = 3). Statistical comparisons were performed against the LAL−/− control group. All p values are Bonferroni-adjusted for multiple comparisons. LAL−/−, lysosomal acid lipase activity-deficient.

P6-13/rscAAV8 improved liver coloration and significantly reduced liver size relative to body weight (Figures 3A,B, 4A,B), and attenuated serum alanine and aspartate aminotransferase levels (Figures 3C,D, 4C,D) in both the young and old cohorts. In the young cohort, all doses of P6-13/rscAAV8 led to marked declines in serum alanine and aspartate aminotransferase compared with the LAL−/− control (p < 0.018; Figures 3C,D). In old cohort, alanine aminotransferase was decreased at the 6 × 10^12^ vg/kg dose (P = 0.006; Figure 4C), and aspartate aminotransferase was decreased at doses of 1 × 10^12^ vg/kg and above (P < 0.026; Figure 4D). For the young cohort, the highest dose of 6 × 10^12^ vg/kg reduced aspartate aminotransferase to 11% of the level in the LAL−/− control (49% higher than in the healthy control). In the old cohort, the same dose reduced aspartate aminotransferase to 20% of the level in the LAL−/− control (2.82-fold higher than in the healthy control).

P6-13/rscAAV8 significantly lowered total cholesterol levels compared to the LAL−/− control (p < 0.001; Figures 3E, 4E) in both the young and old cohorts. At the highest dose, hepatic total cholesterol was 90% of that in the healthy control in the young cohort and 52% higher than in the healthy control in the old cohort. Similarly, P6-13/rscAAV8 significantly decreased cholesteryl ester levels compared with the LAL−/− control (p < 0.004; Figures 3F, 4F) in both cohorts. At the highest dose, hepatic cholesteryl ester level was 57% lower than the healthy control in the young cohort, and 319% higher than the healthy control in the old cohort (Figures 3F, 4F). No significant differences were observed in hepatic triglyceride level between the LAL−/− and healthy control groups in either the young (0.027 ± 0.014 vs. 0.026 ± 0.013 mmol/g liver) or old (0.019 ± 0.0046 vs. 0.024 ± 0.0043 mmol/g liver) cohorts (Figures 3G, 4G).

Oil Red O staining revealed a significant reduction in the lipid-positive area within the livers of P6-13/rscAAV8-treated mice compared with the LAL−/− control (p < 0.001; Figures 3H, 4H). Across all doses, P6-13/rscAAV8 normalized the Oil Red O-positive area to the level observed in healthy controls, except in the old cohort at the low dose (1 × 10^12^ vg/kg), where the area remained 0.32-fold larger. Residual lipid-laden macrophages persisted in all P6-13/rscAAV8-treated mice (Figures 3I, 4I). Consistent with these findings, transmission electron microscopy confirmed that P6-13/rscAAV8 reduced the accumulation of lipid droplets and resolved cholesterol crystals in hepatocytes of the young cohort, and induced the formation of glycogen particles (Figure 3J).

Hematoxylin and eosin staining revealed that P6-13/rscAAV8 treatment attenuated the severity and distribution of inflammatory cell infiltration in the livers of LAL−/− mice in the young cohort, but not in the old cohort, compared with the LAL−/− control (Figures 3I, 4I). Consistent with this, the expression of key pro-inflammatory genes was significantly downregulated in the young cohort at the highest dose (6 × 10^12^ vg/kg) relative to the LAL−/− control (Figure 3K), including *Ccl3* (p = 0.002), *Ccl7* (p = 0.017), *Ccr1* (p = 0.01), *Cd68* (p = 0.002), *Cxcr4* (p = 0.001), *Fcer1g* (p = 0.01), *Tlr7* (p = 0.003), *Tlr8* (p < 0.001), and *Tlr9* (p = 0.018). However, CD68 immunohistochemistry showed no significant difference in macrophage abundance between the P6-13/rscAAV8-treated group and the LAL−/− control in the young cohort (Figure 3L).

Masson staining revealed that P6-13/rscAAV8 treatment, particularly at the highest dose (6 × 10^12^ vg/kg), markedly reduced collagen deposition surrounding macrophages and within perisinusoidal areas in the livers of the young cohort, but not the old cohort (Figures 3M, 4J). Correspondingly, the expression of key pro-fibrogenic genes was significantly downregulated in the young cohort treated with the highest dose compared with the LAL−/− control (Figure 3N), including *Col1a1* (p = 0.019), *Ctgf* (p = 0.025), *Edn1* (p = 0.001), *Lum* (p = 0.007), *Thbs1* (p = 0.030), *Thbs2* (p = 0.031), *Timp1* (p = 0.022), and *Timp2* (p = 0.001).

Transmission electron microscopy suggested that P6-13/rscAAV8 treatment at the highest dose (6 × 10^12^ vg/kg) increased autophagosome formation in the livers of the young cohort compared with the LAL−/− control (Figure 3J). The levels of p62 and LC3-II in both the young and old cohorts were restored to that observed in healthy controls (Figures 3O, 4K). Ultrastructural analysis demonstrated that the highest dose significantly reduced individual mitochondrial area (p = 0.026) and restored mitochondrial ultrastructure, clearing intra-organellar lipid accumulation, in the young cohort compared with the LAL−/− control (Figure 3J). The LAL−/− control exhibited significantly lower mitochondrial complex I activity (1053 ± 900 vs. 2491 ± 1591 U/g liver) and higher complex I and complex IV activity, and ATP levels (Figures 3P-R). Treatment with P6-13/rscAAV8 at the highest dose significantly lowered mitochondrial complex IV activity (p < 0.001; Figure 3Q) and ATP levels (p = 0.001; Figure 3R) in the young cohort compared with the LAL−/− control. Transmission electron microscopy revealed that P6-13/rscAAV8 restored the ultrastructure of endoplasmic reticulum membranes associated with mitochondria. Expression of key endoplasmic reticulum stress genes was significantly downregulated in the young cohort treated with the highest dose compared with the LAL−/− control (Figure 3S), including *Atf4* (p = 0.002) and *Chop* (p = 0.002). Western blot analysis of key endoplasmic reticulum stress related protein revealed higher total eIF2α, and lower phospho-eIF2α and CHOP protein in the LAL−/− control versus the wild-type control. Treatment with P6-13/rscAAV8 partially reversed these alterations (Figure 3T).

### Effects of P6-13/rscAAV8 in the spleen of LAL knockout mice

**FIGURE 5:**
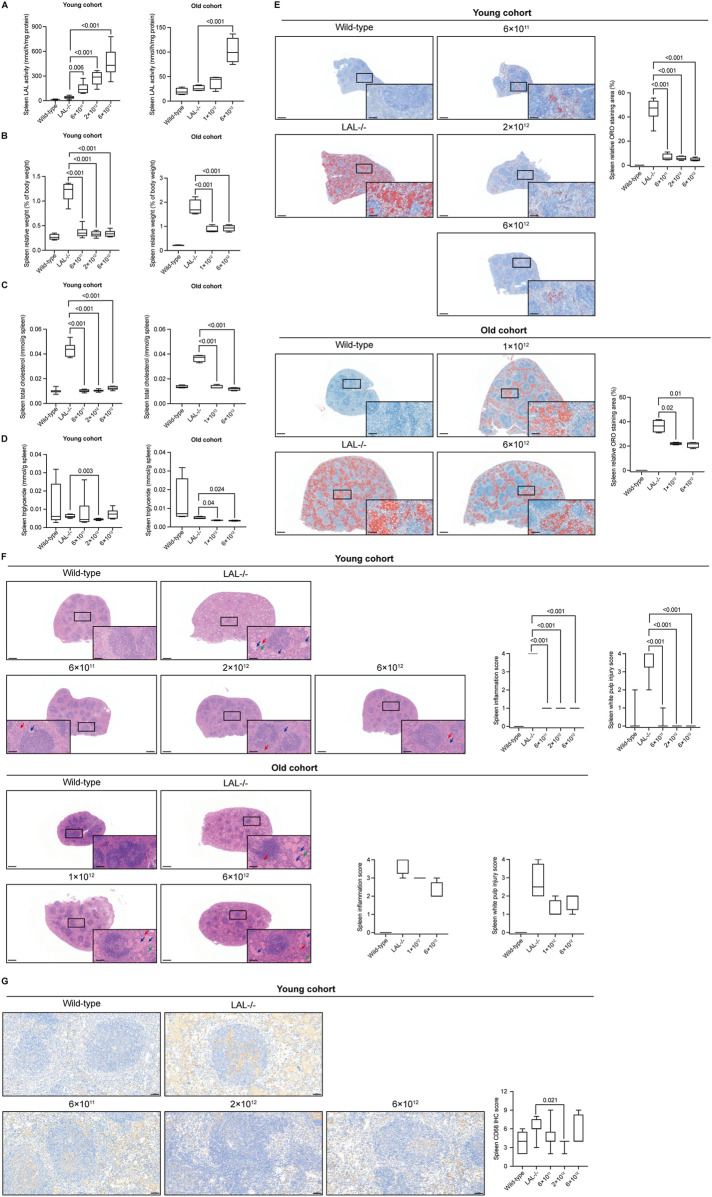
Effects of P6-13/rscAAV8 in the spleen of lysosomal acid lipase activity (LAL) knockout mice. Mice were infected with the virus and treated as described in Figures 1C,D, then the spleens were examined. **(A)** LAL activity. **(B)** Relative spleen weight, expressed as a percentage of body weight. **(C,D)** Levels of total cholesterol and triglyceride in the spleen. **(E,F)** Spleen sections were stained with **(E)** Oil Red O (ORO) or **(F)** hematoxylin-eosin. In hematoxylin-eosin-stained sections, red arrows denote inflammatory cell infiltration, blue arrows denote macrophage hyperplasia with phagocytosed lipids, and green arrows denote extramedullary hematopoiesis. The black boxes (*inset*) are higher-magnification images. Scale bars, 500 μm or 50 μm (inset). **(G)** CD68 was assessed through immunostaining. Scale bar, 50 μm. Statistical comparisons were performed against the LAL−/− control group. All p values are Bonferroni-adjusted for multiple comparisons. IHC, immunohistochemistry; LAL−/−, LAL-deficient. Data are mean ± SD. Young cohort, n = 8; old cohort, n = 4.

We next assessed the therapeutic efficacy of P6-13/rscAAV8 in the spleen. P6-13/rscAAV8 increased LAL activity in a dose-dependent manner, whether in the young or old cohort, and the increase was larger at the earlier stage of disease (10- to 30-fold) than at the later stage (2- to 5-fold; Figure 5A). Similarly, the viral gene therapy restored normal (dark red) coloration of the spleen​ and reduced spleen size in a dose-dependent manner compared to the LAL−/− control (Figure 5B). At the highest dose, the spleen size in the young cohort was reduced to 125% of that in healthy controls, whereas the spleen size in the old cohort remained 336% larger than in healthy controls.

P6-13/rscAAV8 significantly lowered total cholesterol levels in the spleen compared with the LAL−/− control (p < 0.001; Figure 5C) in both cohorts. No significant elevations in triglyceride levels were observed in the spleens of LAL−/− controls in either the young (0.0062 ± 0.00089 vs. 0.012 ± 0.012 mmol/g spleen) or old (0.0050 ± 0.00065 vs. 0.013 ± 0.013 mmol/g spleen) cohorts (Figure 5D). Oil Red O staining revealed a significant reduction in the lipid-positive area within the spleens of P6-13/rscAAV8-treated mice compared with the LAL−/− control (p < 0.02; Figure 5E). At the highest dose, the lipid-positive area in the young cohort was reduced to 11% of that in the LAL−/− control, whereas in the old cohort, it remained 58% of that in the LAL−/− control (Figure 5E). P6-13/rscAAV8 restored white pulp architecture in the spleen in the young cohort, but not in the old cohort, to a state comparable to that observed in healthy controls (p < 0.001; Figure 5F). Similarly, inflammatory cell infiltration and extramedullary hematopoiesis were significantly attenuated at all doses in the young cohort (p < 0.001), but not in the old cohort. Immunostaining against CD68 revealed that macrophage abundance remained largely unchanged following P6-13/rscAAV8 treatment, even at the highest dose in the young cohort (p > 0.05; Figure 5G).

### Effects of P6-13/rscAAV8 on body weight and weights of intestinal segments, iBAT and iWAT of LAL knockout mice

**FIGURE 6:**
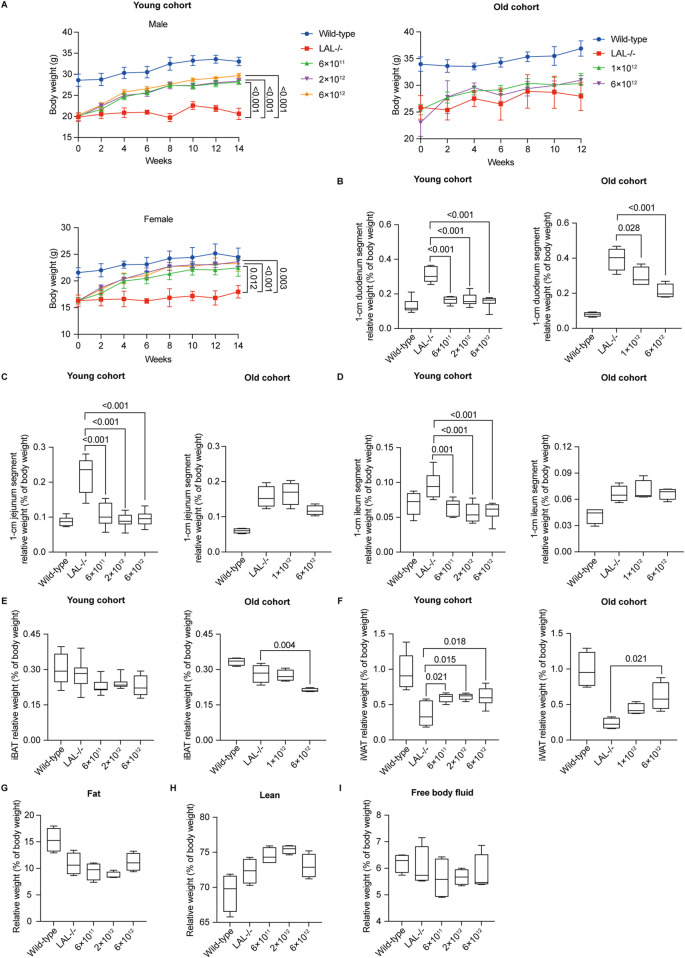
Effects of P6-13/rscAAV8 on body weight, tissue masses, and body composition in lysosomal acid lipase activity (LAL) knockout mice. Mice were infected with the virus and treated as described in Figures 1C,D. Body weight was measured every 2 weeks, and intestinal segments, interscapular brown adipose tissue (iBAT), and inguinal white adipose tissue (iWAT) were harvested at the endpoint. For the young cohort, body composition parameters - including total body fat, lean mass, and fluid - were assessed in awake, non-anesthetized animals immediately prior to euthanasia. **(A)** Body weight during the experiment (n = 4). **(B–D)** Relative tissue mass of 1-cm segments of the **(B)** duodenum, **(C)** jejunum and **(D)** ileum, as well as **(E)** iBAT and **(F)** iWAT as a percentage of body weight (young cohort, n = 8; old cohort, n = 4). **(G–I)** Relative mass of **(G)** fat, **(H)** lean mass, and **(I)** free body fluid (n = 4). Statistical comparisons were performed against the LAL−/− control group. All p values are Bonferroni-adjusted for multiple comparisons. LAL−/−, LAL-deficient.

Higher doses of P6-13/rscAAV8 significantly increased the body weight in the young cohort for both male (p < 0.001) and female (p < 0.012) compared with the LAL−/− control, but not in the old cohort (Figure 6A). Similarly, the virus significantly reduced the weights of 1-cm segments of duodenum (p < 0.001), jejunum (p < 0.001) and ileum (p < 0.001) as a percentage of body weight in the young cohort and to a much weaker extent in the old cohort (Figures 6B–D). In fact, even the highest dose of virus failed to significantly alter the relative weights of the jejunum or ileum in the old cohort (p > 0.05; Figures 6C,D).

The relative weights of iBAT did not differ between healthy controls and LAL−/− controls in the young cohort (0.30% ± 0.065% vs. 0.28% ± 0.061%) (Figure 6E). In contrast, iBAT weight was significantly decreased in the old LAL−/− cohort compared with healthy controls. Treatment with the highest dose of P6-13/rscAAV8 further significantly reduced iBAT weight relative to the LAL−/− control in the old cohort (p = 0.004). Regarding iWAT (Figure 6F), the virus significantly increased its relative weight across all dose groups in the young cohort (p < 0.021) compared with the LAL−/− control. In the old cohort, only the highest dose significantly elevated iWAT weight compared with the LAL−/− control (p = 0.021). Body composition analysis in the young cohort revealed significant alterations in total body fat percentage. Total body fat was significantly decreased in the LAL−/− control compared with healthy controls, and P6-13/rscAAV8 treatment did not significantly alter this parameter relative to the LAL−/− control in the young cohort (p > 0.05; Figure 6G). No significant differences were observed between the LAL−/− and healthy control groups in the young cohort in lean mass (72.32% ± 1.80% vs. 69.31% ± 2.70%) or fluid relative weight (6.03% ± 0.76% vs. 6.21% ± 0.36%) (Figures 6H,I).

### Effects of P6-13/rscAAV8 on LAL activity and lipid profiles in serum of LAL knockout mice

**FIGURE 7:**
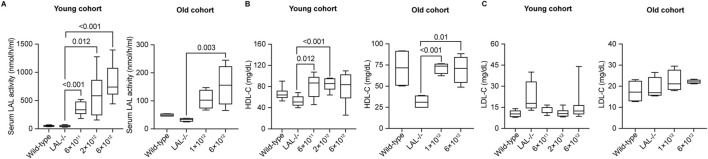
Effects of P6-13/rscAAV8 on lysosomal acid lipase activity (LAL) and lipid profiles in serum of LAL knockout mice. Mice were infected with the virus and treated as described in Figures 1C,D. **(A)** LAL activity. **(B,C)** Levels of high-density lipoprotein cholesterol (HDL-C) and low-density lipoprotein cholesterol (LDL-C). Statistical comparisons were performed against the LAL−/− control group. All p values are Bonferroni-adjusted for multiple comparisons. LAL−/−, LAL-deficient. Data are mean ± SD. Young cohort, n = 8; old cohort, n = 4.

P6-13/rscAAV8 increased lipase activity in serum in a dose-dependent manner (Figure 7A) in both cohorts, but the magnitude of increase was greater in the young cohort (maximally 17-fold vs. 5-fold over LAL−/− controls). Serum HDL-C levels were also significantly higher in both cohorts treated with P6-13/rscAAV8 compared to LAL−/− controls (p < 0.01; Figure 7B). No significant changes were observed in LDL-C levels in serum both in the young and old cohort (p > 0.05; Figure 7C).

### Glycosylation of LAL expressed from P6-13/rscAAV8 in wild-type mice and Huh7 cells

**FIGURE 8:**
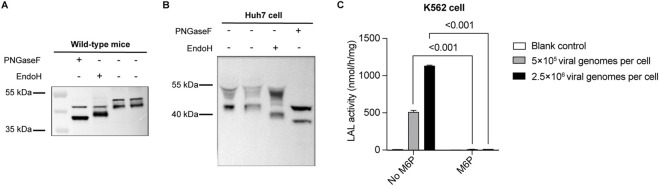
Glycosylation of lysosomal acid lipase (LAL) expressed from P6-13/rscAAV8 in healthy mice and Huh7 cells. **(A)** Mice were treated as shown in Figure 1B. Glycosylation status of LAL in the liver was examined by Western blotting after digestion with PNGase F or Endo H (n = 3). **(B)** Huh7 cells were transduced with P6-13/rscAAV8 at 2.5 × 10^6^ viral genomes per cell; culture medium was collected 1 week later for LAL Western blot analysis after digestion with PNGase F or Endo H (n = 3). **(C)** Huh7 cells were transduced with P6-13/rscAAV8 at 0, 5 × 10^5^ or 2.5 × 10^6^ viral genomes per cell; culture medium was collected 1 week later, and added to K562 cells for 24 h in the presence or absence of 10 mM mannose-6-phosphate (M6P) prior to intracellular LAL activity assay (n = 3). Statistical comparisons were performed against the no-M6P group. All p values are Bonferroni-adjusted for multiple comparisons.

Treatment of liver lysates from wild-type mice and culture medium from P6-13/rscAAV8-infected Huh7 cells with Endo H or PNGase F shifted the migration of LAL on gel electrophoresis (Figures 8A,B), suggesting that the lipase produced from the virally delivered transgene is glycosylated. Endo H cleaves high-mannose and hybrid N-linked oligosaccharides within the chitobiose core, whereas PNGase F removes nearly all N-linked glycans by hydrolyzing the bond between the innermost N-acetylglucosamine and asparagine residues. This glycosylation appeared to be important for lipase activity because the triple-mutant lacking the glycosylatable Arg residues 51, 80 and 300 was expressed at much lower levels within cells and in culture medium (Supplementary Figure 4A), and the resulting enzyme activity was only a few percent of the activity associated with the wild-type transgene (p < 0.001; Supplementary Figure 4B,C).

Conditioned medium from cultures of Huh7 cells infected with P6-13/rscAAV8 increased the activity of LAL within uninfected K562 cells (P < 0.001; Figure 8C), suggesting that exogenous enzyme can enter cells and upregulate the enzyme within host cells. This entry appeared to be mediated by the mannose-6-phosphate receptor because it disappeared in the presence of excess mannose-6-phosphate (p < 0.001; Figure 8C).

## Discussion

Here, we provide evidence that the self-complementary AAV P6-13/rscAAV8 can efficiently induce production of LAL by hepatocytes in a mouse model of lipase deficiency, thereby arresting the histopathology in liver and spleen as well as normalizing systemic lipid metabolism. Notably, P6-13/rscAAV8 restored mitochondrial and endoplasmic reticulum function. These therapeutic effects were much stronger when the virus was administered earlier rather than later in the disease. Our virus system may be able to drive therapeutically effective lipase expression at lower titers than other viral systems, giving it greater clinical potential. In any case, our studies suggest that lipase replacement therapy based on viral delivery of the transgene is more effective when initiated earlier in the disease course.

P6-13/rscAAV8 drove lipase expression in the liver, but not spleen, yet lipase activity increased significantly in both organs and in serum. One possible explanation is that the enzyme is produced and glycosylated in transduced hepatocytes, from which it is secreted into the circulation and then is internalized into spleen cells through a process dependent on the mannose-6-phosphate receptor. Consistent with this, the medium from Huh7 cells transduced with P6-13/rscAAV8 increased LAL activity within K562 cells, and such effect was blocked by either mannose-6-phosphate or mutation of the three known glycosylation sites. In contrast, another study (Rajamohan et al., 2020) found that mutation of the three known glycosylation sites had little effect on enzyme expression and activity. This discrepancy likely reflects the use of different expression systems.

Similar to other models of LAL deficiency and analyses of patients (Grabowski, 2013; Korbelius et al., 2023), homozygous knockout of the enzyme led to massive lysosomal accumulation of cholesteryl esters in hepatocytes as well as in macrophages from liver and spleen, consistent with a role of macrophages in the disorder (Bianco et al., 2023). P6-13/rscAAV8 was not so effective at clearing lipid accumulations within liver and splenic macrophages, despite its ability to normalize lipid levels in hepatocytes. Immunofluorescence analysis of the livers provided direct evidence to support differential efficacy: after P6-13/rscAAV8 administration, a subset of macrophages became smaller and were positive for LAL staining, whereas the remaining large, lipid-laden macrophages lacked detectable LAL signal (Supplementary Figure 5). This limited efficacy in macrophages may be attributed to the liver-specific promoter of P6-13/rscAAV8. This raises a critical consideration for future vector design: while switching to a macrophage-specific promoter would ensure direct transduction, forced overexpression may lead to excessive lysosomal processing that overwhelms cholesterol trafficking capacity, potentially disrupting lysosomal acidification and arresting lysosomal machinery (Dubland and Francis, 2015). Furthermore, extracellular LAL released into the microenvironment might potentiate lipid uptake via scavenger receptors, paradoxically promoting foam cell formation akin to atherosclerotic tissues (Hakala et al., 2003; Haka et al., 2009; Dubland and Francis, 2015). Thus, balancing efficient macrophage targeting with the risk of extracellular spillover or lysosomal overload warrants further investigation.

Beyond the restoration of systemic lipid profiles, P6-13/rscAAV8 treatment ameliorated subcellular pathologies associated with LAL deficiency. Central to this rescue was the mitigation of endoplasmic reticulum stress. Given the established role of LAL in endoplasmic reticulum stress protein maturation (Liu et al., 2022), the observed downregulation of key endoplasmic reticulum stress markers - including *Atf4* and *Chop* - and the restoration of eIF2α phosphorylation pathways suggest that the treatment alleviates the proteotoxic burden imposed by lipid accumulation. The reduction in endoplasmic reticulum stress likely stabilized mitochondrial-associated membranes, thereby preserving mitochondrial homeostasis. Ultrastructural analysis confirmed the restoration of mitochondrial morphology and the clearance of intra-organellar lipid droplets, correlating with the normalization of respiratory chain activities. Specifically, the pathological hyperactivation of Complex IV and ATP production in LAL−/− controls was restored to physiological levels, indicating a shift from inefficient, compensatory respiration back to balanced energy metabolism. Consequently, the resolution of endoplasmic reticulum and mitochondrial dysfunction alleviated the demand for excessive autophagic clearance (He et al., 2023). While transmission electron microscopy revealed an increase in autophagosome abundance, the concurrent restoration of p62 and LC3-II levels to that observed in healthy controls suggests a rebalancing of autophagic flux rather than impairment. Since autophagy is critical for eliminating damaged mitochondria (Chao et al., 2024), its normalization implies that P6-13/rscAAV8 restores the physiological turnover of organelles, preventing the accumulation of toxic debris. Collectively, these findings highlight that promoting cellular homeostasis through the endoplasmic reticulum-mitochondria-autophagy axis is a pivotal mechanism for treating LAL deficiency.

The different intestinal segments contribute differently to lipid absorption, so we examined the effects of P6-13/rscAAV8 administration on the three segments separately. While administration early during disease normalized body weight as well as the weights of duodenum, jejunum and ileum, administration later normalized only the weight of duodenum. Given that the duodenum may be the site of the most severe lipid accumulation in LAL deficiency (Bianco et al., 2023), our observation that late-administered virus did not normalize body weight despite normalizing duodenal weight indicates the importance of addressing lipid accumulation in all segments of the intestine. While these morphological improvements suggest restored organ function, the lack of detailed histopathological analysis represents a limitation in the current study, and future work should assess cellular lipid clearance and tissue architecture across the intestinal tract.

Consistent with the findings by Du et al. (Du et al., 2001), we observed differential susceptibility of adipose tissues to LAL deficiency. While Du et al. demonstrated that BAT remains structurally intact at younger ages but undergoes a pathological transformation into WAT-like tissue with enlarged lipid droplets and reduced thermogenic capacity by 4 months (Du et al., 2001), we observed a preferential loss of iWAT over iBAT in LAL−/− mice, a phenotype that exacerbated with age. P6-13/rscAAV8 treatment restored iWAT weight in a dose-dependent manner in the young cohort, as well as at the highest dose in the old cohort. However, this localized restoration of peripheral fat depots did not translate into significant alterations in total body fat percentage, as measured by whole-body MRI. Such a discrepancy is likely attributable to the anatomical scale of the recovery; iWAT constitutes a relatively minor fraction of total adipose mass, and its repletion may be insufficient to overcome the systemic lipid depletion characteristic of LAL deficiency. Furthermore, while iBAT mass was comparable between LAL−/− and health controls in the young cohort, it was significantly reduced in the old cohort. Notably, treatment with the highest dose of P6-13/rscAAV8 further decreased iBAT weight in old mice compared to the LAL−/− control. This paradoxical reduction suggests that the virus may have reactivated thermogenesis in aged, dysfunctional BAT, leading to increased lipid combustion and a net loss of tissue mass, despite the absence of detectable changes in total body fat or lean mass.

The current study has several limitations. First, the assessment of potential neutralizing antibodies against the AAV8 capsid was not performed, which is a key consideration for re-administration and long-term efficacy. Second, therapeutic efficacy primarily focused on the liver and spleen; we did not perform detailed quantitative histopathological analysis of the intestine, as well as a systematic assessment of other key LAL-expressing tissues (e.g., lung, kidney, adipose tissue). Third, although macrophage lipid content was evaluated, the clearance of lipid accumulations within liver and splenic macrophages appeared less efficient than the robust normalization seen in hepatocytes, highlighting a potential cell-type-specific therapeutic hurdle. Given these points, future studies should focus on: 1) evaluating the long-term safety profile and immune responses in preclinical models; 2) optimizing dosing or vector design to enhance lipid clearance in macrophages; 3) conducting detailed histopathological analyses across all affected tissues; and 4) assessing the optimal therapeutic window for reversing advanced disease. Addressing these challenges will be crucial for the successful translation of this approach into possible use in human patients.

In summary, the current study showed that P6-13/rscAAV8 can strongly induce hepatic expression of functional LAL and reverse most of the histopathology associated with LAL deficiency. These findings encourage further studies of P6-13/rscAAV8 in phase I/II clinical trials.

## Data Availability

The original contributions presented in the study are included in the article/Supplementary Material, further inquiries can be directed to the corresponding author.
