# Supplementary material for: Liver-specific gene therapy based on self-complementary adeno-associated virus for lysosomal acid lipase deficiency
Source: Front Pharmacol. 2026 Jul 1;17:1822727. doi: 10.3389/fphar.2026.1822727 (PMC13368558; doi:10.3389/fphar.2026.1822727)
Supplement: Supplementary file 1 [file Supplementaryfile1.docx]

**Supplementary Table 1 List of primers used in the study**

| Target gene | Primer | Sequence 5’-3’ |
| --- | --- | --- |
| transgene-specific *LIPA* | LIPA-F | TGATCAAGGACCTGTTCGGC |
|  | LIPA-R | GAAACTTCACGGCTTGGCTC |
| 18s rRNA | 18s rRNA-F | AGAAACGGCTACCACATCCA |
|  | 18s rRNA-R | CCCTCCAATGGATCCTCGTT |
| *Col1a1* | Col1a1-F | GCTCCTCTTAGGGGCCACT |
|  | Col1a1-R | CCACGTCTCACCATTGGGG |
| *Col3a1* | Col3a1-F | CCTGGCTCAAATGGCTCAC |
|  | Col3a1-R | CAGGACTGCCGTTATTCCCG |
| *Col6a2* | Col6a2-F | GCTCCTGATTGGGGGACTCT |
|  | Col6a2-R | CCAACACGAAATACACGTTGAC |
| *Ctgf* | Ctgf-F | GGGCCTCTTCTGCGATTTC |
|  | Ctgf-R | ATCCAGGCAAGTGCATTGGTA |
| *Edn1* | Edn1-F | GCACCGGAGCTGAGAATGG |
|  | Edn1-R | GTGGCAGAAGTAGACACACTC |
| *Itga11* | Itga11-F | TGCCCCAATGGAAACCAATG |
|  | Itga11-R | CACTCGTGCGACCAGAGAG |
| *Lum* | Lum-F | CTCTTGCCTTGGCATTAGTCG |
|  | Lum-R | GGGGGCAGTTACATTCTGGTG |
| *Tgfb2* | Tgfb2-F | TCGACATGGATCAGTTTATGCG |
|  | Tgfb2-R | CCCTGGTACTGTTGTAGATGGA |
| *Thbs1* | Thbs1-F | GGGGAGATAACGGTGTGTTTG |
|  | Thbs1-R | CGGGGATCAGGTTGGCATT |
| *Thbs2* | Thbs2-F | CTGGGCATAGGGCCAAGAG |
|  | Thbs2-R | GCTTGACAATCCTGTTGAGATCA |
| *Timp1* | Timp1-F | GCAACTCGGACCTGGTCATAA |
|  | Timp1-R | CGGCCCGTGATGAGAAACT |
| *Timp2* | Timp2-F | TCAGAGCCAAAGCAGTGAGC |
|  | Timp2-R | GCCGTGTAGATAAACTCGATGTC |
| *Ccl3* | Ccl3-F | TTCTCTGTACCATGACACTCTGC |
|  | Ccl3-R | CGTGGAATCTTCCGGCTGTAG |
| *Ccl7* | Ccl7-F | GCTGCTTTCAGCATCCAAGTG |
|  | Ccl7-R | CCAGGGACACCGACTACTG |
| *Ccr1* | Ccr1-F | CTCATGCAGCATAGGAGGCTT |
|  | Ccr1-R | ACATGGCATCACCAAAAATCCA |
| *Cd68* | Cd68-F | ACACTTCGGGCCATGTTTCT |
|  | Cd68-R | GGGGCTGGTAGGTTGATTGT |
| *Cxcl1* | Cxcl1-F | CTGGGATTCACCTCAAGAACATC |
|  | Cxcl1-R | CAGGGTCAAGGCAAGCCTC |
| *Cxcr4* | Cxcr4-F | GACTGGCATAGTCGGCAATG |
|  | Cxcr4-R | AGAAGGGGAGTGTGATGACAAA |
| *Fcer1g* | Fcer1g-F | ATCTCAGCCGTGATCTTGTTCT |
|  | Fcer1g-R | ACCATACAAAAACAGGACAGCAT |
| *Il18rap* | Il18rap-F | AGACTACTTCCTGAGCACAAGA |
|  | Il18rap-R | TGTCCTTACCAATGGTTCTCACT |
| *Tlr7* | Tlr7-F | ATGTGGACACGGAAGAGACAA |
|  | Tlr7-R | GGTAAGGGTAAGATTGGTGGTG |
| *Tlr8* | Tlr8-F | GAAAACATGCCCCCTCAGTCA |
|  | Tlr8-R | CGTCACAAGGATAGCTTCTGGAA |
| *Tlr9* | Tlr9-F | ATGGTTCTCCGTCGAAGGACT |
|  | Tlr9-R | GAGGCTTCAGCTCACAGGG |
| *Bip* | Bip-F | CAGCCAATTATCAGCAAACTCT |
|  | Bip-R | CAACTCCACTCTGAGGTGAAG |
| *Atf4* | Atf4-F | CCTTCGACCAGTCGGGTTTG |
|  | Atf4-R | CTGTCCCGGAAAAGGCATCC |
| *Atf6* | Atf6-F | GAAGACTGGGAGTCGACGTT |
|  | Atf6-R | ACTCCCAAGGCATCAAATCCAA |
| *Chop* | Chop-F | CGGAACCTGAGGAGAGAGTG |
|  | Chop-R | GTCTCCAAGGTGAAAGGCAG |
| *Xbp1* | Xbp1-F | CCGCAGCACTCAGACTATGT |
|  | Xbp1-R | GTTCCTCCAGACTAGCAGACTC |
| *Traf2* | Traf2-F | AAGCGTCAGGAAGCCGTAG |
|  | Traf2-R | CATTCAAGTAGACTCGTAGACACAT |
| *Caspase12* | Caspase12-F | TCCTCAGACAGCACATTCCT |
|  | Caspase12-R | TTCTCAGACTCCGACAGTTAGA |

**
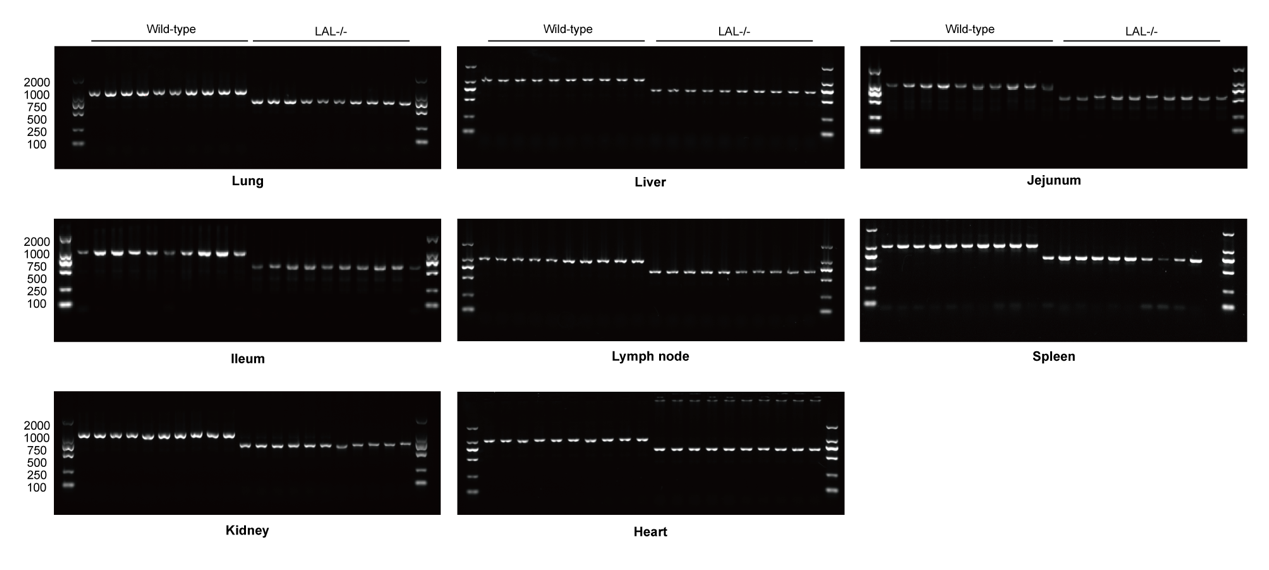
Supplementary Figure 1. Verification of LAL gene knockout in multiple tissues of LAL knockout mice. ​**Genomic DNA was extracted from lung, liver, jejunum, ileum, lymph node, spleen, kidney, and heart tissues of wild-type (wild-type) and LAL knockout (LAL-/-) mice. PCR amplification was performed using primers targeting the LAL gene locus (forward: 5’-CAGGATGGACTGACACAGGC-3’; reverse: 5’-GCAGGGGGAGGCTAGTTTTT-3’). Expected amplicon sizes were 1066 bp for the wild-type allele and 717 bp for the knockout allele. Bands of the expected size were detected in all tissues examined, confirming the absence of the wild-type LAL allele and systemic knockout of the LAL gene. The knockout was generated by CRISPR/Cas9-mediated deletion of exon 3 using the following guide RNAs: gRNA1: 5’-GCCTGCGAGTATAACTGACTGGG-3’, gRNA2: 5’-AGTTACCACAAGACGGCTGGAGG-3’. n = 10.

**
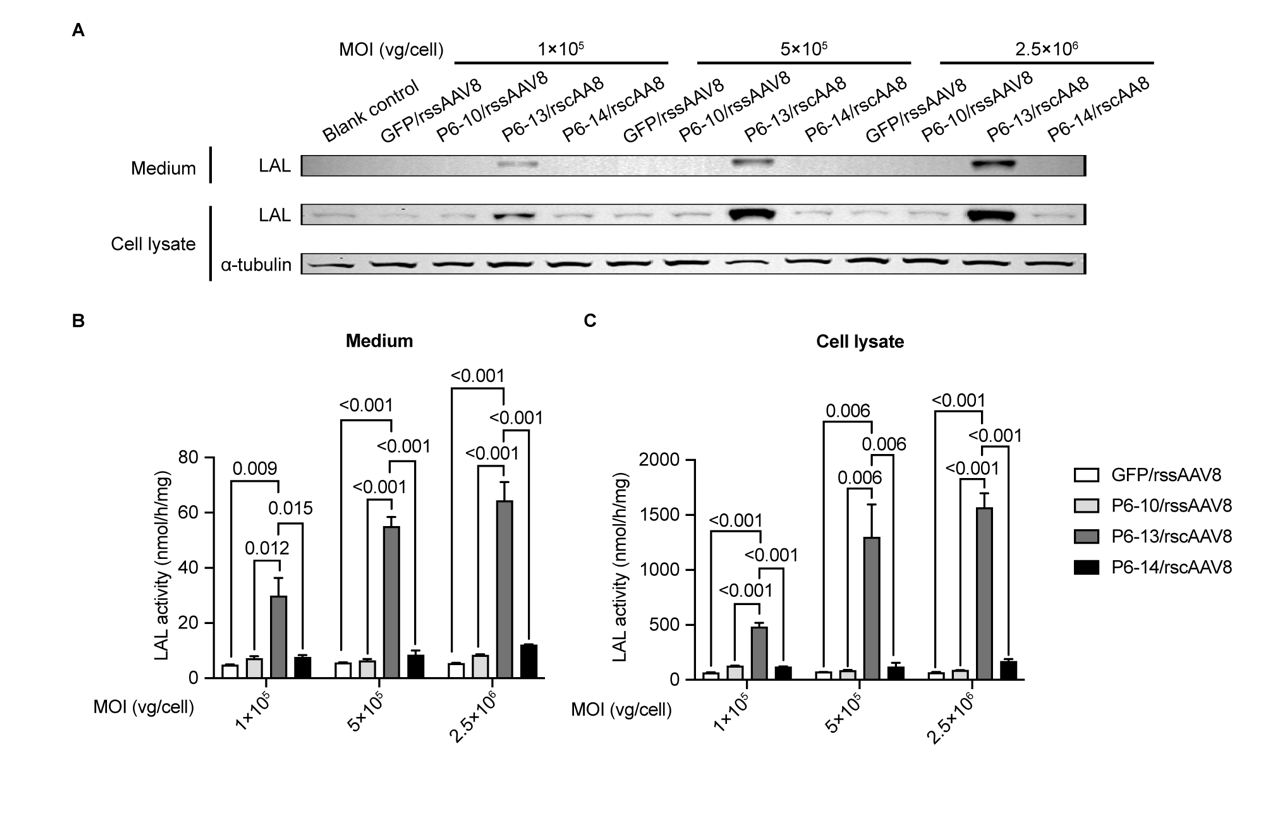
Supplementary Figure 2. Comparison of expression and activity of lysosomal acid lipase (LAL) driven by different adeno-associated viruses in Huh7 cells.** Huh7 cells were transduced with viruses differing in their translational regulatory elements controlling the LAL transgene (P6-10/rssAAV8, P6-13/rscAAV8, P6-14/rscAAV8) or with the control single-stranded virus encoding green fluorescent protein (GFP/rssAAV8) at the indicated multiplicities of infection (MOIs). At one week after transduction, culture medium and cell lysate were analyzed. **(A)** Representative Western blot. **(B-C)** LAL activity. n = 3. Data are mean + SD. Statistical comparisons were performed against the P6‑13/rscAAV8​ group at the corresponding MOI. All p values are Bonferroni-adjusted for multiple comparisons; p < 0.05 was considered statistically significant. vg, viral genome.

**
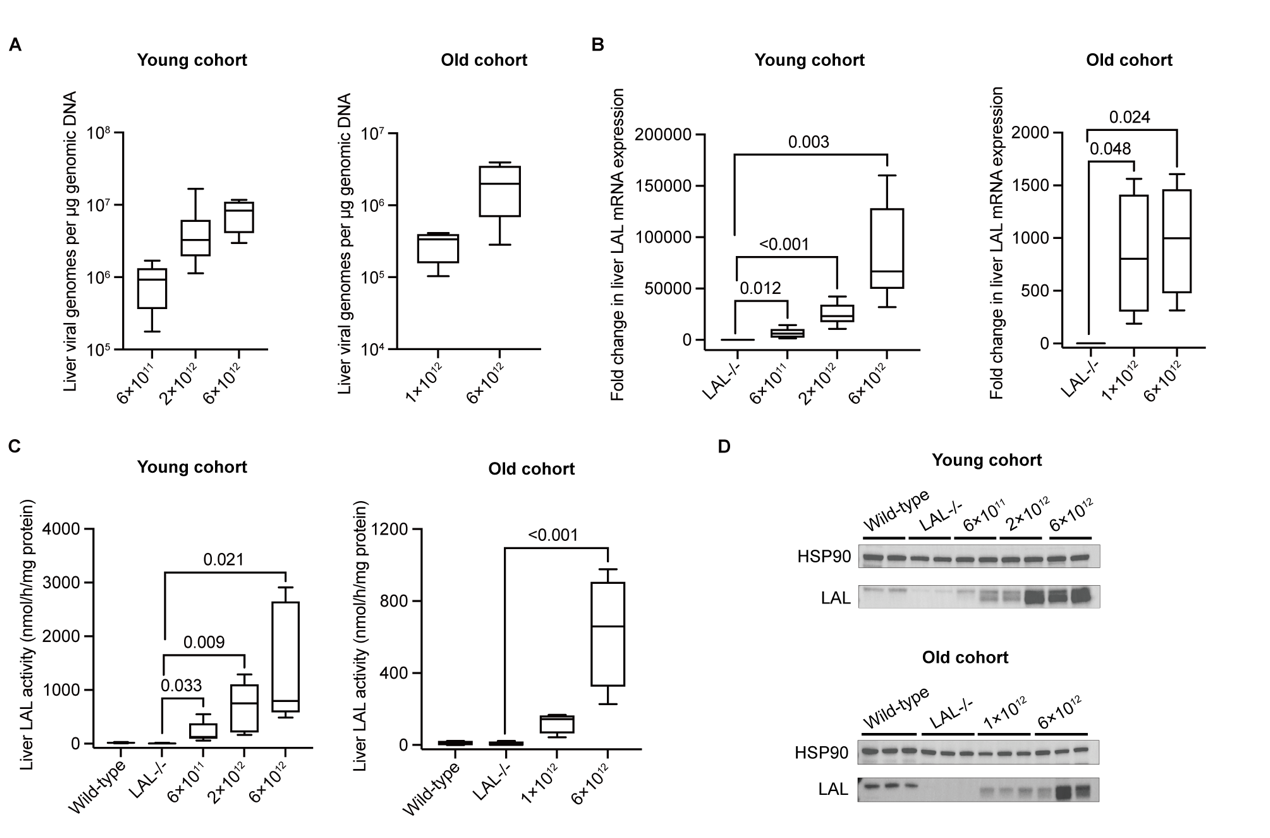
Supplementary Figure 3. Replication and expression of P6-13/rscAAV8 in the liver of lysosomal acid lipase knockout mice (LAL-/-).** Mice with homozygous LAL knockout received a single intravenous injection of P6-13/rscAAV8 at the indicated virus doses (viral genomes per kg) at 9 weeks old (young cohort; n = 4 per group for each sex) and at 28 weeks old (old cohort; n = 4 per group, all male). Healthy mice and LAL-/- mice infected with control virus encoding green fluorescent protein (GFP) at 6 × 10^12^ viral genomes per kg were included as controls. Mice were euthanized at 14 weeks for young cohort or 13 weeks for old cohort after infection. **(A)**​ Viral genome copies per μg of genomic DNA in LAL‑/- mice treated with different doses of P6‑13/rscAAV8 (young cohort, n = 8; old cohort, n = 4) **(B)**​ Fold change in LAL mRNA levels in P6‑13/rscAAV8‑treated LAL‑/- mice relative to LAL‑/- controls (young cohort, n = 8; old cohort, n = 4) **(C)**​ LAL enzymatic activity (young cohort, n = 8; old cohort, n = 4) **(D)**​ LAL protein levels assessed by Western blotting (n = 3); heat‑shock protein 90 (HSP90) was used as a loading control for Western blot. The anti‑LAL antibody recognizes both murine and human LAL. Statistical comparisons were performed against the LAL‑/- control group. All p values are Bonferroni‑adjusted for multiple comparisons; p < 0.05 was considered statistically significant.


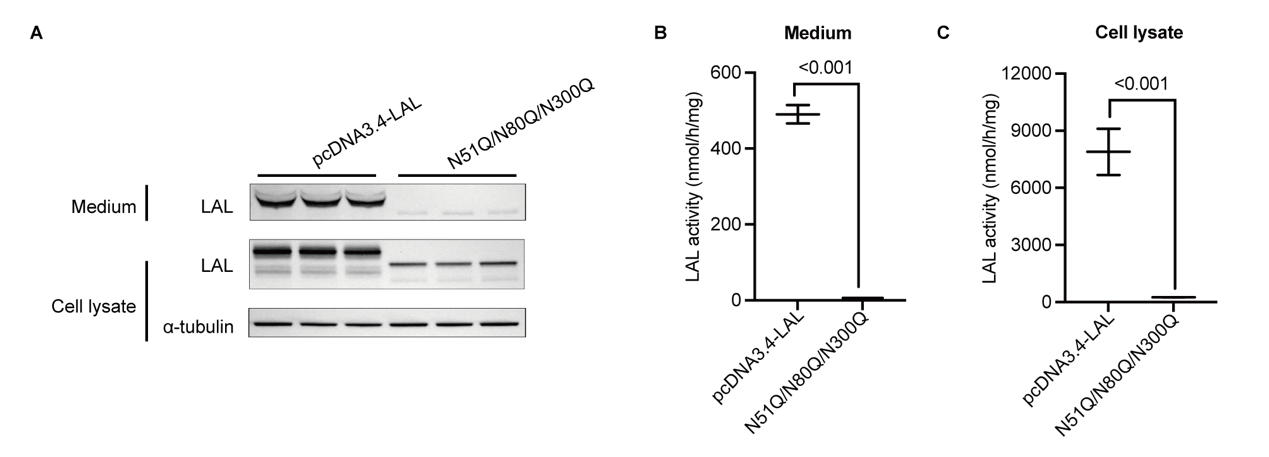
**Supplementary Figure 4. Effect of N-linked glycosylation on expression and activity of lysosomal acid lipase (LAL) in Huh7 cells.** Huh7 cells were transfected with plasmids encoding wild-type LAL (pcDNA3.4-LAL) or a triple mutant of LAL lacking three known glycosylation sites (N51Q/N80Q/N300Q). At 48 h after transfection, culture medium and cell lysates were analyzed. **(A)** Representative Western blots. α‑Tubulin was used as a loading control. **(B-C)** LAL activity. n = 3. Statistical comparisons between groups were performed using a Student’s *t*‑test. P < 0.05 was considered statistically significant.


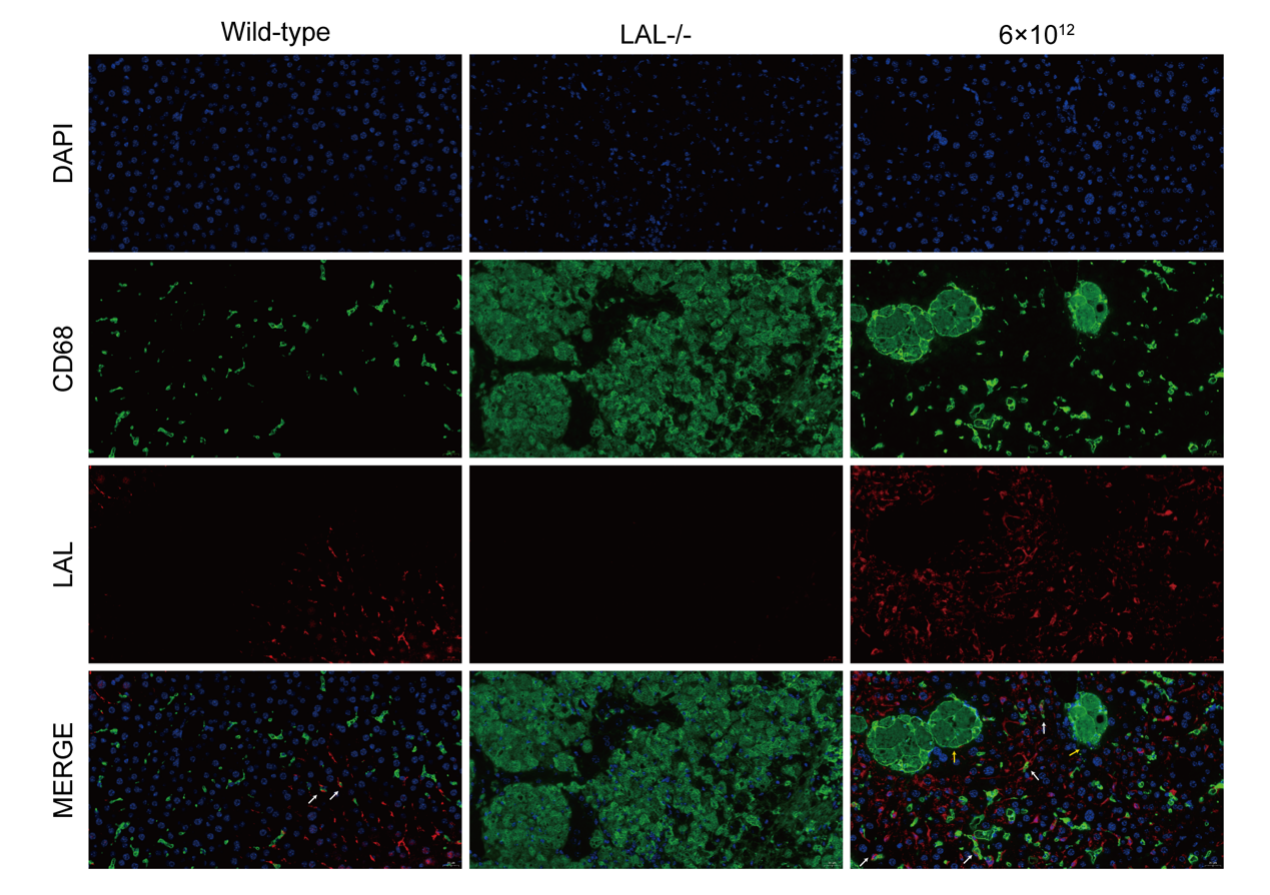


**Supplementary Figure 5. Co-localization of lysosomal acid lipase (LAL) and cluster of differentiation 68 (CD68) in mouse liver sections.** Mice with homozygous LAL knockout (LAL-/-) received a single intravenous injection of P6-13/rscAAV8 at the 6 × 10^12^ viral genomes per kg at 9 weeks old (n = 4 per group for each sex). Healthy mice and LAL-/- mice infected with control virus encoding green fluorescent protein (GFP) at 6 × 10^12^ viral genomes per kg were served as controls. Mice were euthanized at 14 weeks after infection. Liver sections were processed for sequential tyramide signal amplification immunofluorescence (TSA-IF): LAL (iF555, red), CD68 (iF488, green), and DAPI (blue). Scale bar = 20 µm. White arrows indicate small CD68^+^ macrophages exhibiting LAL co-localization; yellow arrows indicate large, lipid-laden CD68^+^ macrophages lacking detectable LAL signal. n = 8.
